# *Stomoxys calcitrans* as a potential mechanical vector of *Anaplasma phagocytophilum*: assessment through original *ex vivo* feeding models

**DOI:** 10.1051/parasite/2026021

**Published:** 2026-04-22

**Authors:** Clotilde Rouxel, Guillem Weis-Servat, Emmanuel Liénard, Shukri Sharif, Anne-Claire Lagrée, Pierre Lucien Deshuillers, Henri-Jean Boulouis, Nadia Haddad, Emilie Bouhsira

**Affiliations:** 1 ANSES, INRAE, Ecole Nationale Vétérinaire d’Alfort, Laboratoire de Santé Animale, BIPAR Maisons-Alfort F-94700 France; 2 Université de Toulouse, ENVT, INRAE, InTheRes Toulouse France; 3 Libyan Biotechnology Research Center Tripoli Libya

**Keywords:** *Anaplasma phagocytophilum*, *Stomoxys calcitrans*, Stable flies, Mechanical vector, *Ex vivo* infection model

## Abstract

Granulocytic anaplasmosis is a zoonotic disease that affects various domestic mammals (dogs, horses, and, more rarely, cats). In ruminants, it is better known as tick-borne fever (TBF) and is responsible for significant economic losses on European livestock farms, mainly due to a drop in milk production, abortions, and immunosuppression, which can lead to secondary infections. The disease is caused by the strictly intracellular bacterium *Anaplasma phagocytophilum*, whose biological vectors are ticks of the genus *Ixodes*. Other blood-feeding arthropods may be involved in transmitting this bacterium, notably *Stomoxys calcitrans*, a major ectoparasite of livestock that is implicated in transmitting other pathogens, including bacteria of the genus *Anaplasma*. This study aimed to evaluate the potential of *S. calcitrans* to act as a mechanical vector of *A. phagocytophilum* under laboratory conditions. Two experimental models were employed: one mimicking immediate transmission, and the other delayed transmission. In both models, *A. phagocytophilum* DNA and RNA were detected in *S. calcitrans* for the first time, but no traces of the bacterium’s DNA or RNA were found in the glass feeder’s blood. Further research is needed to confirm these findings through field studies investigating the presence of the bacterium in flies under natural conditions. This study also describes two original infection models of stable flies designed to reproduce their *ex vivo* blood-feeding, promoting alternative experimental approaches in accordance with animal welfare regulations and 4R principles.

## Abstract (fr)

L’anaplasmose granulocytaire est une maladie zoonotique pouvant toucher divers mammifères domestiques (chiens, chevaux et plus rarement le chat). Chez les ruminants, cette maladie plus connue sous le nom de *Tick-Borne Fever* (TBF), est responsable d’importantes pertes économiques dans les élevages européens en raison notamment de chutes de production laitière, d’avortements et d’immunodépressions pouvant conduire à des infections secondaires. Cette maladie est causée par *Anaplasma phagocytophilum*, bactérie intracellulaire stricte dont les vecteurs biologiques sont les tiques du genre *Ixodes*. D’autres arthropodes hématophages pourraient être impliqués dans la transmission de cette bactérie, notamment *Stomoxys calcitrans,* ectoparasite important des bovins incriminé dans la transmission d’agents pathogènes, dont des bactéries du genre *Anaplasma*. L’objectif de cette étude a été d’évaluer le rôle de *S. calcitrans* en tant que vecteur mécanique potentiel d’*A. phagocytophilum* en conditions de laboratoire. Deux modèles expérimentaux ont été utilisés, l’un mimant une transmission immédiate et l’autre mimant une transmission retardée. Dans ces deux modèles, la présence d’ADN et d’ARN d’*A. phagocytophilum* a été détectée pour la première fois dans *S. calcitrans*, cependant aucune trace d’ADN ou d’ARN de la bactérie n’a été détectée dans le sang du receveur. Il serait intéressant de confirmer ces données par des études de terrain en recherchant la présence de la bactérie dans les mouches en conditions naturelles. Cette étude présente également deux modèles originaux d’infection des stomoxes permettant de reproduire leur prise de repas sanguins *ex vivo*, et promeut ainsi des approches expérimentales alternatives respectueuses de l’éthique animale, conformément à la règle des 4R.

## Introduction

Granulocytic anaplasmosis (GA) is a zoonotic disease caused by a strictly intracellular bacterium, *Anaplasma phagocytophilum*, which multiplies mainly in neutrophils. Its biological vectors are ticks of the genus *Ixodes*, mainly *Ixodes ricinus*, also known to be the most common tick species found on cattle in Europe [29].

GA has significant human and animal health consequences. Highly prevalent in the United States, human GA is steadily increasing with more than 60,000 cases reported between 2008 and 2023 in this country [7]. GA can also affect several domestic mammal hosts, including dogs and horses [29]. Only a few cases in cats have been reported in the literature [34]. It is also responsible for major economic losses in ruminants in Europe, where it is more commonly known as tick-borne fever (TBF). TBF is characterized by fever, hyperthermia, reduced milk production, and lameness [10]. In France, according to the Observatory of the causes of abortion in ruminants (OSCAR) network, 26.2% of abortions recorded in cattle in 2023 (among the cases of bovine abortion that were investigated) could be attributed to TBF alone, or in combination with another abortive disease [28]. In addition, secondary infections, in particular tick pyaemia due to *Staphylococcus aureus*, may benefit from the immunodepression observed in ruminants suffering from TBF [29, 44].

In addition to ticks, various haematophagous arthropods (flies, *Culicoides*, mosquitoes, etc.) can infest domestic ruminants and transmit pathogens of veterinary importance [18]. Among them, *Stomoxys calcitrans* (Diptera: Muscidae), also commonly known as the stable fly, is considered to be the main pest in cattle [20]. *Stomoxys calcitrans* is found worldwide and the infestation level in cattle is high, reaching up to 1,000 adult flies per animal per day during the vector activity season [31]. In the USA, economic losses caused by stable flies in the meat and dairy industries were estimated by the United States Department of Agriculture (USDA) to be approximately 2.4 billion dollars per year [39], while, in France, these losses were estimated to range from 145 and 234 million euros annually [3]. *Stomoxys calcitrans* can have a significant impact on livestock health and welfare, both directly and indirectly. Firstly, its repeated painful bites are responsible for stress, energy loss, reduced feed intake, skin lesions, blood loss, and reduced immunity, leading in particular to a reduction in weight gain and milk yield [25]. Secondly, *S. calcitrans* has an indirect effect as a mechanical vector for several pathogens of veterinary significance, including viruses (lumpy skin disease virus, equine infectious anaemia, bovine leukosis virus, etc.) [1] and parasites (e.g. *Besnoitia besnoiti*, *Trypanosoma evansi*, and *Trypanosoma vivax*) [19, 21, 36]. They are also suspected of being vectors of another bacterium of the genus *Anaplasma*, *Anaplasma marginale* [2, 24, 30]. The mechanical transmission of pathogens by *S. calcitrans* remains poorly understood. Two modes of transmission have been proposed based on the fly’s behaviour. The first one is immediate, sequential transmission between two hosts after an interrupted blood meal, typically due to defensive host movements caused by the pain of the bite. When *S. calcitrans* resumes its meal on the second host, pathogens can be transferred through the inoculation of residual infected blood remaining on the mouthparts. In this case, the mouthparts may act as a contaminated needle. The second suggested transmission mode is delayed transmission, possibly occurring through the regurgitation of the infected blood from the crop during a subsequent blood meal. In contrast to direct transmission, which tends to involve animals in close proximity (“intra-herd” transmission), delayed transmission may facilitate the spread of pathogens between animals located farther apart, such as individuals from neighbouring herds (“inter-herd” transmission) [1, 31]. In fact, in 2010, in a study carried out by Taylor *et al.* [38], the majority of *S. calcitrans* adults (50%) dispersed beyond 1.6 km from their larval development site, but only 5% dispersed beyond 5.1 km. These distances may vary, depending on various factors (weather conditions, host distribution and activity, topography, etc.), and the maximum local displacement of *S. calcitrans* has been estimated to be 13 km [37]. However, a study conducted in Belgium showed that the dispersal potential of *S. calcitrans* is affected by the repletion stage, with maximum flight distances of 150 m and 300 m for partially and unfed *S. calcitrans*, respectively. It has been hypothesised that unfed *S. calcitrans* have a more urgent need to take a blood meal and may therefore travel longer distances to find a host [17].

To date, the biological transmission of *A. phagocytophilum* has only been demonstrated in ticks, and there is a lack of evidence of mechanical transmission by haematophagous dipterans [9]. The aim of the present study was to investigate the potential role of *S. calcitrans* in the mechanical transmission of *A. phagocytophilum* under experimental conditions. Two innovative *ex vivo* models were used, mimicking the potential feeding behaviour of stable flies in the field, as previously described [19]. Firstly, an interrupted blood meal model was used to assess potential immediate transmission of *A. phagocytophilum* between two artificial feeders. Secondly, a complete blood meal model was used to monitor the persistence of *A. phagocytophilum* in *S. calcitrans* over time after infection and to determine whether delayed transmission could occur in such conditions.

## Material and methods

### Culture of the *A. phagocytophilum* NY-18 isolate

The American human NY-18 isolate of *A. phagocytophilum* was grown in HL-60 cells (240-CCL; ATCC, Manassas, VA, USA), as previously described [15]. Cell infection was determined using Hemacolor^®^ staining (Merck Millipore, Molsheim, France) after cytocentrifugation. All *A. phagocytophilum* cultures were carried out under BSL-2 conditions, at the UMR BIPAR laboratory (ANSES, INRAE, EnvA) of Maisons-Alfort, France. The *A. phagocytophilum*-infected cells were then sent, at room temperature, to the École Nationale Vétérinaire de Toulouse (ENVT), France, in less than 24 h and used immediately.

### Stable fly colony

The flies used in this study originated from a colony maintained by the Parasitology Department at the ENVT in France since 2009 as described by Salem *et al*. [32]. Briefly, adult stable flies, males and females, are maintained in mesh cages in a temperature- and humidity-controlled environment (22 ± 2 °C; 40 ± 10 %) and a 12:12 light-dark cycle. They are fed daily with cattle blood using an artificial feeding system and allowed to shed eggs on the bottom of the breeding cages. Eggs are collected daily and left to evolve in a larval medium for approximately 3 weeks. The mean life cycle from egg to adult is 19.2 ± 1.7 days [32].

### Experimental design

**Figure 1:**
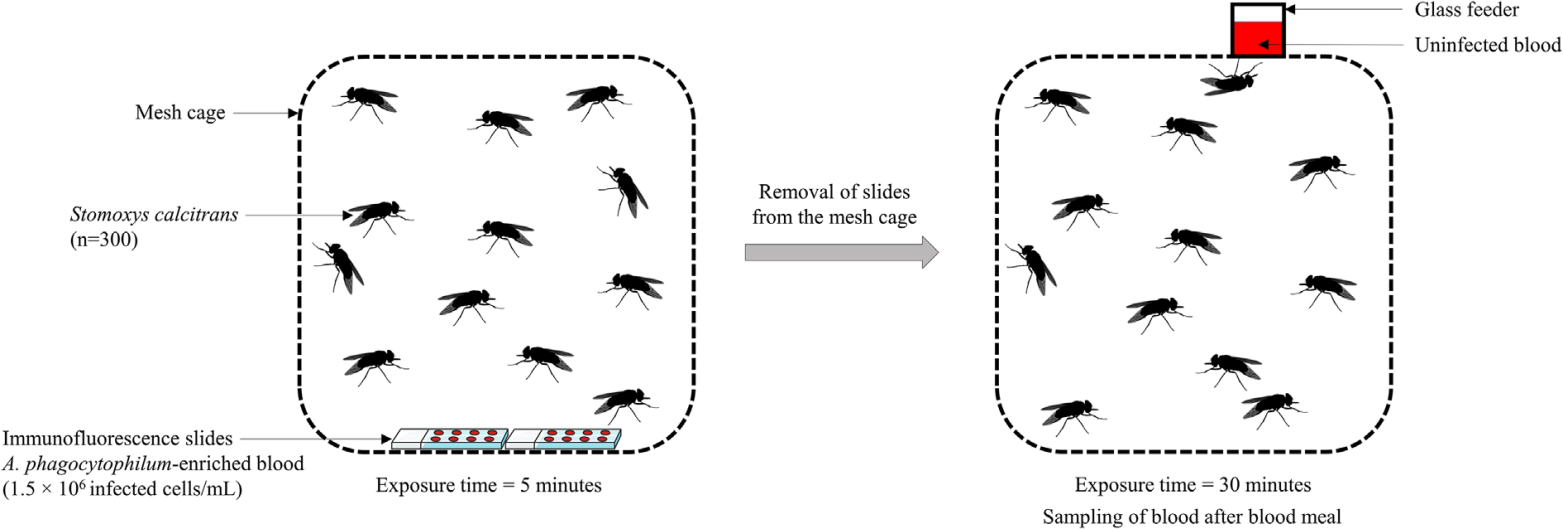
Diagram illustrating the experimental design of the interrupted blood meal (schematic frontal view). *Stomoxys calcitrans* were exposed to *Anaplasma phagocytophilum*-enriched blood on immunofluorescence slides, which were removed from the mesh cage after 5 min, followed by a 30-min uninfected blood meal provided through a glass feeder. Blood was sampled at the end of the blood meal.

**Figure 2:**
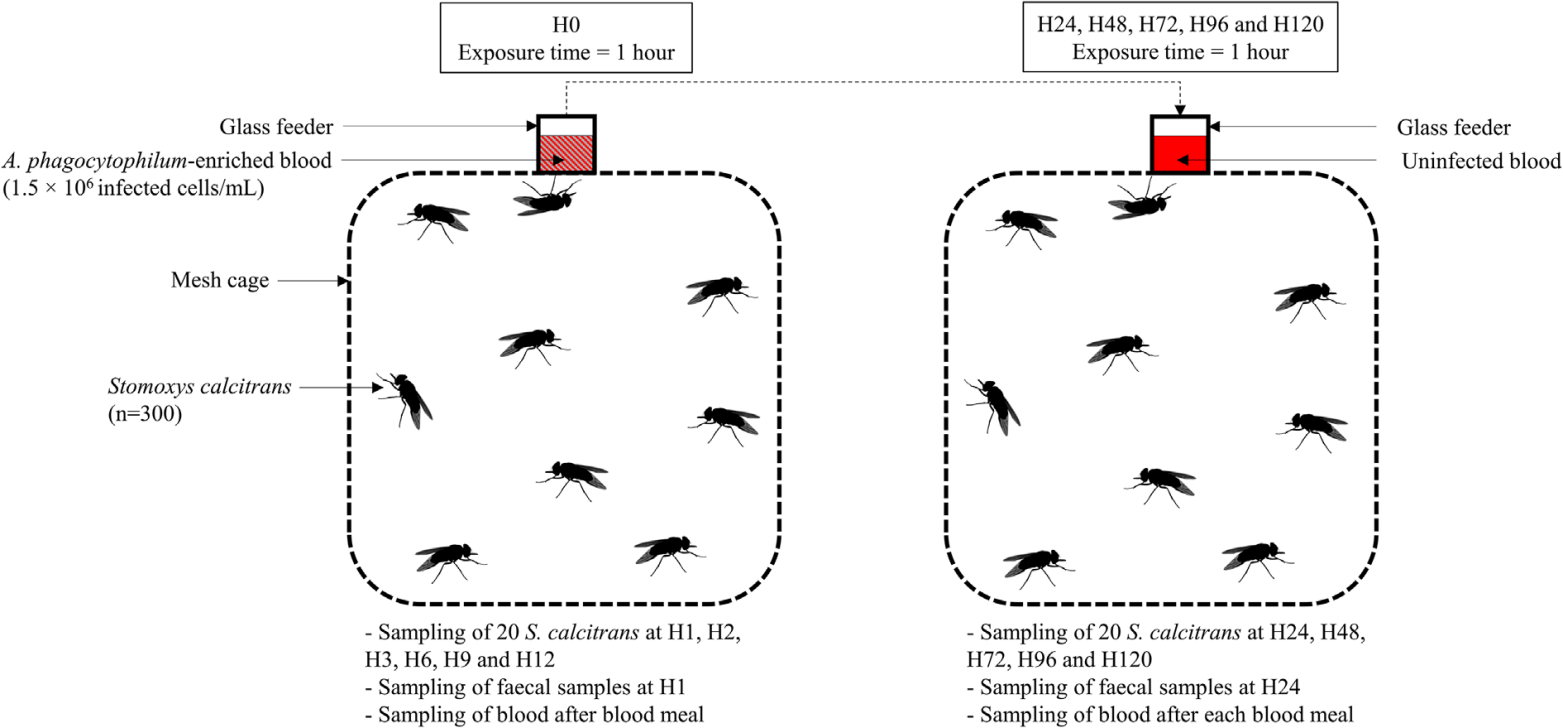
Diagram illustrating the experimental design of the complete blood meal (schematic frontal view). *Stomoxys calcitrans* were exposed to *Anaplasma phagocytophilum*-enriched blood provided through a glass feeder at H0 for 1 hour. Subsequently, every 24 h, flies were exposed to an uninfected blood meal through a glass feeder for 1 h. Batches of 20 flies were collected from the mesh cage at H1, H2, H3, H6, H9, H12, H24, H48, H96, and H120. Faecal samples were collected from the mesh cage at H1 and H24. Blood was collected at the end of each blood meal.

For each of the two experiments (interrupted (IntM) and complete (ComM) blood meal), four batches of approximately 300 (±10 flies) male and female flies (sex-ratio 1:1) aged between 1 and 2 days were used and transferred from the colony into four mesh cages (30 × 30 × 30 cm) 5 days before each experiment. Three batches were exposed to infected cattle blood (“infected batches”), while 1 batch of 300 flies was exposed to uninfected cattle blood (control batch). They were fed with uninfected cattle blood 96 h, 72 h and 48 h before each infection. The four batches were conducted in parallel. They were provided with honey and water *ad libitum*. These two experiments were carried out twice, with (i) batches 1, 2, 3 and control, and (ii) batches 4, 5, 6 and control (Table 1; Figs. 1 and 2).

**Table 1:** Design of experiments. A batch control was performed with batches 1, 2, and 3 and also with batches 4, 5, and 6.

		Interrupted blood meal (IntM)	Complete blood meal (ComM)
		Batch numbers	Batch numbers
DNA	*S. calcitrans*	1, 2, 3, 4, 5, 6	4, 5, 6
	Blood	4, 5, 6	4, 5, 6
	Faeces	*N.D.*	4, 5, 6
RNA	*S. calcitrans*	1, 2, 3	1, 2, 3
	Blood	1, 2, 3	1, 2, 3

*N.D.* not determined.

### Infection of blood with *Anaplasma phagocytophilum* suspension

Cattle blood was sourced from residual clinical samples collected at the ENVT large animal hospital. All infections were performed at the ENVT insectarium. Upon reception at ENVT, *A. phagocytophilum* suspensions were centrifuged at 2,000× *g* for 10 min. The supernatant was discarded and the pellet resuspended in 6 mL of uninfected cattle blood at a concentration around 1.5 million infected cells per mL. The suspension was then used immediately for the subsequent infection of stable flies.

### Interrupted blood meal

An amount of 20 μL of uninfected blood (control batch), or blood infected with *A. phagocytophilum* were placed in each 8 mm well of 6 immunofluorescence slides (IF) (76 × 26 mm) (Labelians, Nemours, France) heated to 38 °C for 30 min on electric hotplates. The total amount of blood was deliberately insufficient for full engorgement of all flies. IF slides were placed into the mesh cage for 5 min (insufficient time for the flies to fully engorge) and then replaced by an artificial glass feeder containing uninfected cattle blood, for 30 min so that flies could complete their blood meal. A temperature of 38.5 °C was maintained by a water jacket system that circulated water through the glass feeder to mimic the host’s body temperature and stimulate the flies to feed. Then flies were placed at −20 °C for 30 min, separated into batches of 20 individuals and stored at −80 °C until further analysis. The glass feeder was also removed and the remaining blood was collected and stored at −80 °C (Fig. 1).

### Complete blood meal

Batches of 300 flies were exposed to an artificial glass feeder, which was placed on top of the mesh cage, closed at the bottom with 2 thin Parafilm M^®^ membranes (Pechiney Plastic Packaging, Chicago, IL, USA) and filled with 6 mL of uninfected (control batch) or infected cattle blood. A temperature of 38.5 °C was maintained by a water jacket system that circulated water through the glass feeder. The flies were exposed to the blood for one hour. Then the glass feeder was removed and the remaining blood was collected and stored at −80 °C for further analysis. The time point H0 was defined at the starting time of the feeding. Batches of 20 flies were then sampled at H1, H2, H3, H6, H9, H12, H24, H48, H72, H96, and H120 then stored at −80 °C. Moreover, after each collection time at H24, H48, H72, H96, and H120, the flies were exposed to uninfected cattle blood and left to feed for 1 h. At the end of each blood meal, the blood remaining in the glass feeder was collected and stored at −80 °C. At H120, all remaining flies were placed at −80 °C until analysed (Fig. 2).

Additionally, to confirm the presence of *A. phagocytophilum* in the stable fly, faecal samples were collected at 1 h and 24 h post-feeding using five sterile swabs moistened with 1X sterile PBS (Bio Basic Inc., Markham, ON, Canada). Swabs were passed over all the walls of the cage in order to collect as much faeces as possible. The swabs were then immersed individually in 500  μL of sterile PBS for 5 min before being stored at −80 °C for further analysis.

All biological samples were transferred at −80°C to the UMR BIPAR laboratory (ANSES, INRAE, EnvA) of Maisons-Alfort for molecular biology analysis.

## Detection of *A. phagocytophilum* in *S. calcitrans* and blood samples

### DNA extraction of *S. calcitrans*, blood, and faecal samples

DNA extraction was performed using a NucleoSpin^®^ Tissue Kit (Macherey-Nagel, Düren, Germany).

Each *S. calcitrans* was placed separately in a 1.5 mL Eppendorf tube containing 180 μL of T1 lysis buffer and 6 stainless steel beads (Ozyme, Saint-Cyr-l’École, France), then mechanically disrupted using a Precellys^®^24 Dual Homogenizer (Bertin Technologies, Montigny-le-Bretonneux, France) at 5,500 rpm for 20 s. Centrifugation at 1,500× *g* for 30 min was then performed and the supernatant transferred to a 1.5 mL Eppendorf tube. For the lysis step, 25 μL of Proteinase K was added to each sample prior to incubation at 56 °C for 3 h at 850 rpm. DNA was then extracted, according to the manufacturer’s instructions. DNA was eluted in a final volume of 50 μL.

For blood samples, 25 μL of Proteinase K and 200 μL of Buffer B3 were added to 200 μL of blood. After a 5-min incubation at room temperature, the mixture was incubated at 70 °C for 30 min at 800 rpm. The supplier’s instructions were then followed. DNA was eluted in a final volume of 60 μL.

Faecal samples were centrifuged at 4,000× *g* for 15 min. The resulting pellet was then resuspended in 200 μL of T1 lysis buffer. DNA extraction was then carried out in accordance with the manufacturer’s instructions and eluted in a final volume of 50 μL.

A NanoDrop spectrophotometer (Thermo Fisher Scientific, Waltham, MA, USA) was used to determine DNA concentration and quality using absorbance ratios of 260/280 nm and 260/230 nm.

### DNA preamplification and detection of *A. phagocytophilum* DNA with *msp2* Taqman qPCR

*Anaplasma phagocytophilum* DNA was detected in *S. calcitrans* and blood samples by Taqman real-time PCR targeting a 77 bp region of the gene encoding major surface protein 2 (*msp2*) [8].

Initially, pre-amplification was performed on samples with low expected levels of *A. phagocytophilum* DNA (blood samples except H0 and H1 time). In a final volume of 5 μL, 1.25 μL of DNA was added to a mix containing 1 μL of PreAmp Master mix (Standard Biotools, San Francisco, CA, USA), 1.25 μL of a pool containing 0.2 μM of forward (5′–ATG GAA GGT AGT GTT GGT TAT GGT ATT–3′) and reverse (5′–TTG GTC TTG AAG CGC TCG TA–3′) *msp2* primers (Eurofins Genomics, Ebersberg, Germany), and sterile distilled water. Pre-amplification was performed using a thermocycler (Eppendorf, Montesson, France) with the following conditions: 95 °C for 2 min, followed by 14 cycles of amplification at 95 °C for 15 s and 60 °C for 4 min. PCR products obtained were diluted fivefold in ultra-pure water and stored at −20 °C.

qPCR was performed in a LightCycler 480 thermocycler (Roche, Basel, Switzerland). A mix of 2 μL of DNA, 6 μL of LightCycler 480 Probes Master 2× (Roche, Basel, Switzerland), 0.2 μM of forward and reverse *msp2* primers, 0.2 μM of probe (5′–TGG TGC CAG GGT TGA GCT TGA GAT TG–3′) and sterile water q.s. 12 μL was performed for TaqMan qPCR. Thermocycler cycle conditions were as follows: pre-incubation for 10 min at 95 °C, followed by 45 cycles of amplification, with the following parameters: 10 s at 95 °C and 15 s at 60 °C.

Positive and negative controls were included in each experiment. The negative control was ultra-pure water, and the positive control was DNA from HL-60 cells infected with *A. phagocytophilum* NY-18.

### RNA extraction from *S. calcitrans* and blood samples

Total *S. calcitrans* RNA was extracted using a NucleoSpin^®^ RNA Kit (Macherey-Nagel, Düren, Germany), according to the manufacturer’s instructions. Each fly was placed in an Eppendorf tube containing 6 stainless steel beads, 350 μL of RA1 buffer, and 3.5 μL of β-Mercaptoethanol (MP Biomedicals, Irvine, CA, USA). Samples were then homogenized using a Precellys^®^ 24 Dual Homogenizer at 5,500 rpm for 20 s. Centrifugation at 15,000× *g* for 5 min at 4 °C was then carried out before continuing with the following steps. Elution was performed in a 40 μL volume.

Extraction of total RNA from blood was performed using the NucleoSpin^®^ RNA Blood Kit (Macherey-Nagel, Düren, Germany), according to the manufacturer’s instructions. Elution volume was 60 μL.

DNase treatment of RNA to eliminate contaminating genomic DNA was then carried out using the Turbo DNA-free^TM^ kit (Thermo Fisher Scientific), according to the manufacturer’s instructions. RNA was stored at −80° C.

A NanoDrop spectrophotometer was used to determine the concentration and quality of the RNA before and after DNase treatment using absorbance ratios at 260/280 nm and 260/230 nm.

### Reverse transcription

Total RNA was reverse transcribed using SuperScript^TM^ III First-Strand Synthesis SuperMix (Thermo Fisher Scientific). Amounts of 200 ng to 1 μg of total RNA were annealed with random hexamers (50 ng) in the presence of 1 μL annealing buffer and RNase/DNase-free water q.s. 8 μL at 65 °C for 5 min, followed by at least 1 min at 4 °C. Primer extension was then performed by adding 1× first strand reaction mix and 2 μL SuperScript^TM^ III / RNAseOUT^TM^ enzyme mix to a final volume of 12 μL. This mix was incubated at 25 °C for 10 min and then at 50 °C for 50 min. The reaction was terminated at 85 °C for 5 min and cDNA was then stored at −20 °C. Negative controls without reverse transcriptase enzyme were prepared for all samples.

### cDNA pre-amplification and *msp2* Taqman qPCR

A pre-amplification step was then performed on the cDNAs (except blood samples at H0 time), followed by a Taqman qPCR *msp2* using the same conditions as above.

### *rps3* qPCR

The *rps3* (ribosomal protein S3) gene of *S. calcitrans* was amplified by qPCR as an internal control to verify the presence of amplifiable RNA [23]. Primers were designed using Primer3Plus (https://www.primer3plus.com/; [41]). qPCR was run with a LightCycler 480 thermocycler using a mixture consisting of 2 μL of cDNA, 10 μL of LightCycler 480 SYBRGreen 2X (Roche, Basel, Switzerland), 0.5 μM of forward (*rps3*-F 5′–CTT CGA ACC TGG CCG TAT TG–3′) and reverse (*rps3*-R 5′–ACA CAA CAA CTT CAC AGC CC–3′) primers and sterile water q.s. 20 μL. Thermal cycling conditions consisted of an initial denaturation step at 95 °C for 10 min, followed by 45 cycles of amplification: 10 s at 95 °C, 10 s at 58 °C, and 15 s at 72 °C. To verify the specificity of the reaction, melting curve analyses were performed. Positive and negative controls were included in each experiment. The negative control was ultra-pure water, and the positive control was RNA from HL-60 cells infected with *A. phagocytophilum* NY-18.

## Results

The presence of bacteria in the blood at H0 was confirmed by the detection of *A. phagocytophilum* DNA and RNA in all experiments (both interrupted and complete feeding models) with Ct values ranging from 13.94 to 17.20 and from 20.93 to 27.07 for DNA and RNA, respectively (Supplementary Table 1).

### Study of the immediate mechanical transmission of *A. phagocytophilum* by *S. calcitrans* using the interrupted feeding model

#### Detection of *A. phagocytophilum* DNA and RNA in *S. calcitrans* after an interrupted infected blood meal

**Figure 3:**
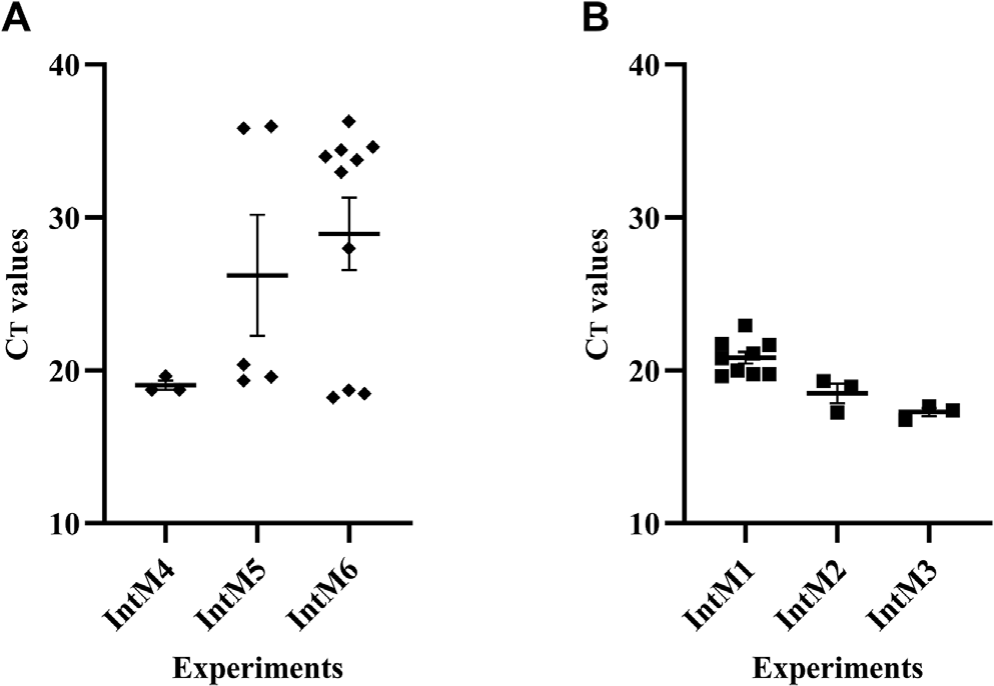
Detection of *Anaplasma phagocytophilum* DNA (without pre-amplification) (A) and RNA (with pre-amplification) (B) in *Stomoxys calcitrans* after an interrupted meal of infected blood. Ten *S. calcitrans* were tested per experiment (*n* = 3) and the mean Ct ± SEM was calculated on positive flies (IntM: interrupted blood meal).

**Supplementary Figure 1.**
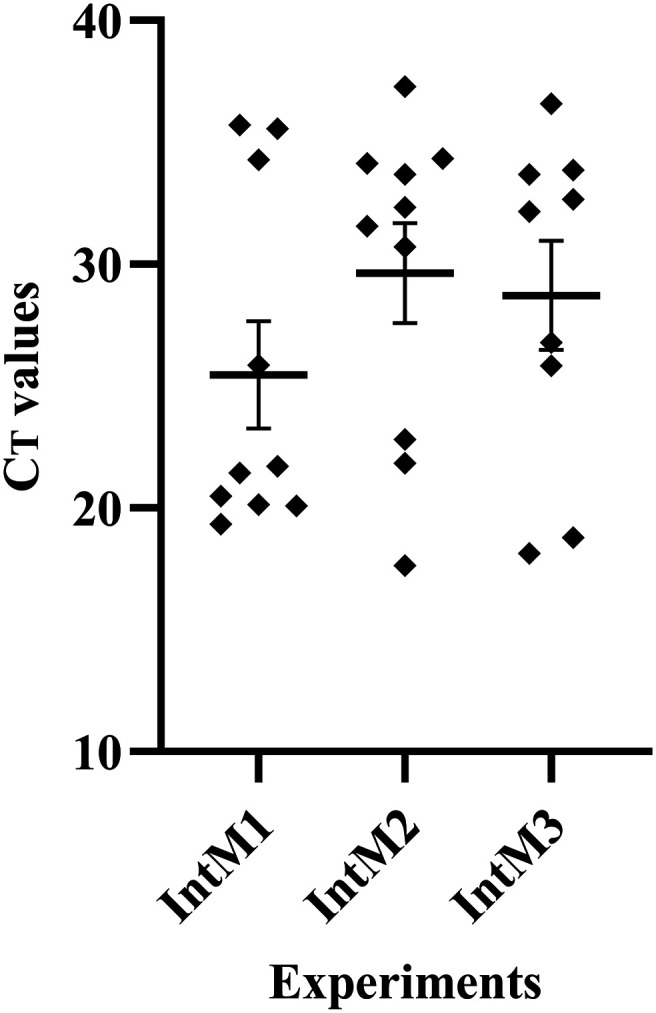
Detection of *Anaplasma phagocytophilum* DNA in *Stomoxys calcitrans* after an interrupted meal of infected blood.

Following an interrupted infected blood meal, the presence of *A. phagocytophilum* DNA (experiments IntM1 to IntM6) was detected in *S. calcitrans*, with mean Ct values ranging from 19.04 to 29.63. Positive *S. calcitrans* rates of 3/10 and 5/10 (experiments IntM4 and IntM5, respectively), 9/10 (IntM3), and 10/10 (IntM1, IntM2 and IntM6) were observed, with a higher proportion of positive *S. calcitrans* for 4/6 replicates (Fig. 3A; Table 2 and Supplementary Figure 1). Similarly, *A. phagocytophilum* RNA was detected in *S. calcitrans*, with mean Ct values ranging from 17.28 to 20.83 (Fig. 3B; Table 2). One replicate (IntM1) showed a higher number of positive flies (9/10), while in the other two replicates (IntM2 and IntM3), the number of positive individuals was only 3/10. It is important to note that, in these experiments (IntM1, IntM2, and IntM3), a much higher proportion of *S. calcitrans* tested positive for *A. phagocytophilum* DNA (10/10, 10/10, and 9/10) than in previous experiments (Table 2 and Supplementary Figure 1).

**Table 2:** Detection of *A. phagocytophilum* DNA and RNA in *S. calcitrans* after an interrupted meal of infected blood.

	DNA detection		RNA detection^#^	
Experiments	Mean^*^ Ct ± SEM	Positive *S. calcitrans* for Aph *msp2*	Mean^*^ Ct ± SEM	Positive *S. calcitrans* for Aph *msp2*
IntM1	25.46 ± 2.20	10/10	20.83 ± 0.38	9/10
IntM2	29.63 ± 2.06	10/10	18.50 ± 0.64	3/10
IntM3	28.72 ± 2.24	9/10	17.28 ± 0.27	3/10
IntM4	19.04 ± 0.30	3/10	*N.D.*	*N.D.*
IntM5	26.22 ± 3.96	5/10	*N.D.*	*N.D.*
IntM6	28.94 ± 2.38	10/10	*N.D.*	*N.D.*

^*^Mean Ct on Aph-positive *S. calcitrans*.

^**#**^With pre-amplification.

*N.D.* not determined.

Aph: *A. phagocytophilum.*

IntM: Interrupted blood meal.

### Detection of *A. phagocytophilum* DNA and RNA in the blood in the glass feeder after an interrupted infected blood meal

Neither *A. phagocytophilum* DNA nor RNA were detected in the blood in the glass feeder, even after pre-amplification.

### Study of delayed mechanical transmission of *A. phagocytophilum* by *S. calcitrans* using the complete feeding model

#### Detection of *A. phagocytophilum* DNA and RNA in *S. calcitrans* after a complete meal of infected blood

**Figure 4:**
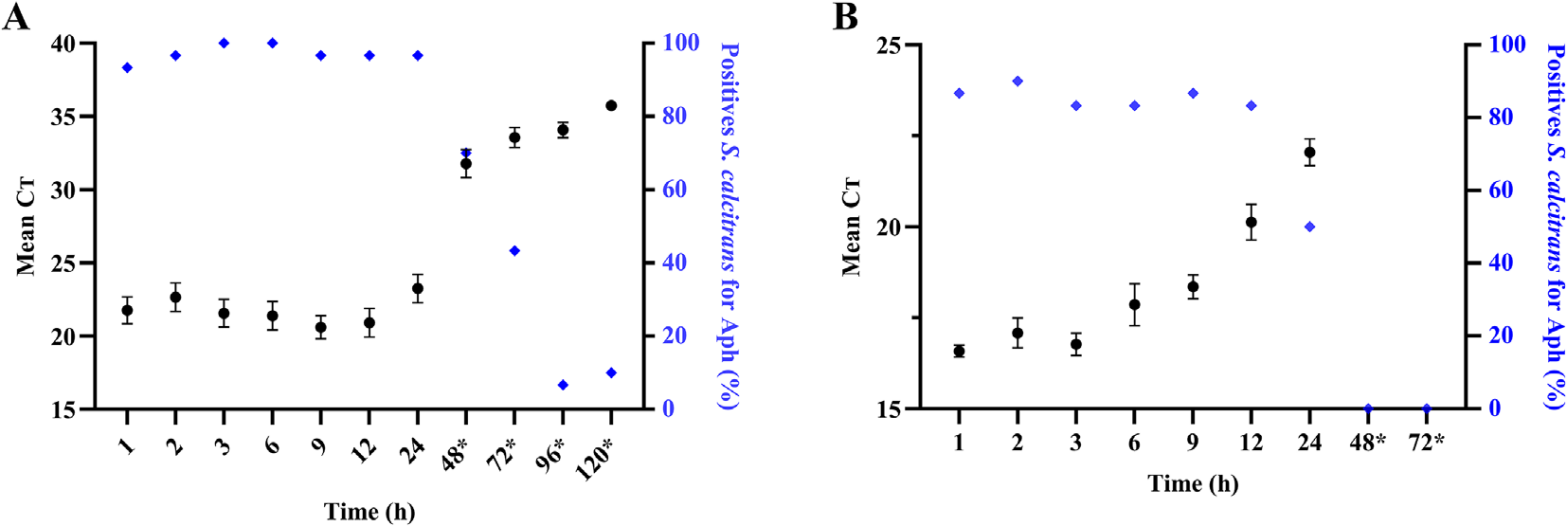
Detection of *Anaplasma phagocytophilum* DNA (without pre-amplification) (A) and RNA (with pre-amplification) (B) at different times in *Stomoxys calcitrans* after a complete meal of infected blood. Thirty *S. calcitrans* were tested per time (ten *S. calcitrans* per experiment (*n* = 3)) and the mean Ct ± SEM was calculated on positive flies. *Flies were fed with uninfected blood every 24 h starting 48 h after the initial blood meal. *Aph: A*. phagocytophilum.

**Supplementary Figure 2.**
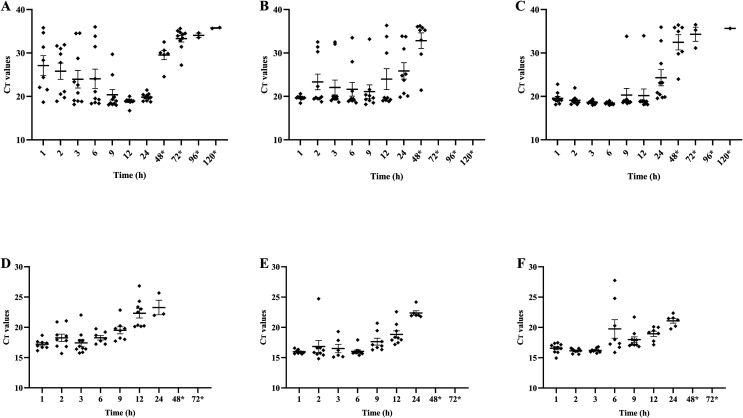
Detection of *Anaplasma phagocytophilum* DNA and RNA at different times in *Stomoxys calcitrans* after a complete meal of infected blood.

*Anaplasma phagocytophilum* DNA was detected in *S. calcitrans* with relatively low mean Ct values (experiments ComM4, ComM5, and ComM6), ranging from 20.59 to 23.25, up to 24 h after a complete meal of infected blood. Furthermore, a high number of *S. calcitrans* tested positive for *A. phagocytophilum* (>28/30). A significant increase of Ct values (31.77 up to 35.73) was then observed from 48 hours to 120 hours after the infected blood meal. In the same way, a significant decrease in the number of *A. phagocytophilum*-positive *S. calcitrans* was also observed between 48 hours (21/30 *S. calcitrans* positives) and 120 hours with only 3/30 *S. calcitrans* positives (Fig. 4A; Supplementary Figure 2A, 2B, 2C and Supplementary Table 2A).

*Anaplasma phagocytophilum* RNA was present in *S. calcitrans* up to 24 h after the infected blood meal with mean Ct values (experiments ComM1, ComM2 and ComM3) ranging from 16.58 to 22.05. The number of *A. phagocytophilum*-positive flies remained almost consistent, ranging between 25/30 and 27/30, from time H1 to H12. However, a 50% decline in *A. phagocytophilum* RNA levels in positive individuals was noted 24 h after feeding on infected blood. No RNA was detected in the flies at 48 h and 72 h in three replicates (Fig. 4B; Supplementary Figure 2D, 2E, 2F and Supplementary Table 2B).

#### Detection of *A. phagocytophilum* DNA and RNA in the blood in the glass feeder after a complete meal of infected blood

No DNA was detected in the blood from the glass feeder, 24, 48, 72, 96, and 120 h after a complete meal of infected blood in the three replicates. In the same way, no RNA was present in the blood from the glass feeder after 24 h, in the three replicates.

#### Detection of *A. phagocytophilum* DNA and RNA in faeces after a complete meal of infected blood

Detection of *A. phagocytophilum* DNA in faeces was observed 1 h and 24 h after a complete meal of infected blood with mean Ct values of 32.88 and 33.70, respectively (Table 3).

**Table 3:** Ct values of *msp2* qPCR observed in the faeces of *S. calcitrans* 1 h and 24 h after a complete meal of infected blood.

	Time after feeding	
	1 h	24 h
ComM4	35.10	34.98
ComM5	32.95	34.38
ComM6	30.59	31.75
Mean Ct ± SEM	32.88 ± 1.30	33.70 ± 0.99

ComM: Complete blood meal.

## Discussion

This study investigates, for the first time, the potential role of *S. calcitrans* in the transmission of *A. phagocytophilum* using innovative *ex vivo* models reproducing feeding behaviour in natural conditions. An interrupted feeding model was used to determine whether immediate transmission occurs, while a complete feeding model was used to monitor the presence of *A. phagocytophilum* in *S. calcitrans* over time, assessing the possibility of delayed mechanical transmission through blood regurgitation. Artificial feeding of laboratory-reared *S. calcitrans* has previously been employed to study the mechanical transmission of other pathogens and has proven to be an effective approach. For instance, this approach has been used to investigate the transmission of the protozoan *Besnoitia besnoiti* [19] and the Lumpy Skin Disease Virus [26, 33] by *S. calcitrans*. The current study reports, for the first time, an interrupted blood meal infection model that aligns with the behaviour of stable flies in the field.

*Anaplasma phagocytophilum* is an intracellular bacterium that infects neutrophils, which have a very limited lifespan (less than 24 h when used *ex vivo* in culture) [4]. However, it has been demonstrated that *A. phagocytophilum* can be transmitted via blood transfusion and can survive for over a week in leukoreduced red blood cell units stored at 4 °C [40]. Therefore, even if, *ex vivo*, blood is an unfavourable environment for *A. phagocytophilum* to survive and multiply, this could only partially explain the absence of DNA and RNA detection in the blood in the glass feeder.

In order to achieve optimal conditions (i.e. conditions mimicking the highest infection load reported in naturally infected cattle), the bacteraemia level in the blood at time H0 was relatively high, with Ct values ranging from 13 to 17 for both models. These levels are not very different from those reported in some naturally-infected cattle. A study conducted in Germany with PCRs that used the same primers (*msp2*) and probe sequences on blood samples collected from cows showed Ct values of around 18–19. However, some Ct values were significantly higher (32–33) [22].

This study showed the presence of *A. phagocytophilum* DNA and RNA in *S. calcitrans* for the first time, using an interrupted and complete feeding model. However, these data should be confirmed by field-investigation studies, since the detection of *A. phagocytophilum* DNA or RNA in *S. calcitrans* like in any other blood-sucking insects does not necessarily indicate that these insects are mechanical vectors. Moreover, it would be interesting to culture the infected *S. calcitrans* homogenate; however, culturing *A. phagocytophilum* is complex, which makes infectivity testing difficult. For example, *A. phagocytophilum* has also been detected in the field, in several other blood-sucking insects. In Poland, *A. phagocytophilum* DNA was found in three species within the Tabanidae (*Haematopota pluvialis*, *Tabanus bromius*, and *Tabanus distinguendus*) with prevalence rates of 24% [43]. *Anaplasma phagocytophilum* DNA was also found in deer keds (*Lipoptena cervi*) collected from cervids in Slovakia (*Cervus elaphus* and *Capreolus capreolus*) [42] and in the USA (*Odocoileus virginianus*), [27] with 10.5% and 30% prevalence rates, respectively. Finally, *A. phagocytophilum* DNA was found in 2% of the mosquitoes tested (*Aedes albopictus*, *Armigeres subalbatus*, and *Culex tritaeniorhynchus*) in China [11]. However, none of these insects are currently considered vectors of *A. phagocytophilum*, and demonstrating the role of blood-sucking insects such as *S. calcitrans* as mechanical vectors can be challenging. For example, despite several investigations, the role of *S. calcitrans* in the transmission of *A. marginale* remains unclear. Several studies have found *A. marginale* DNA in *S. calcitrans*, even in areas where ticks are absent [2, 30]. However, two experimental studies using different models reported unsuccessful transmission of *A. marginale* to cattle by *S. calcitrans* [13, 35].

Under natural conditions, mechanical transmission in the context of an interrupted meal notably depends on the volume of residual blood on the insects’ mouthparts [1]. Therefore, although *A. phagocytophilum* DNA and RNA were detected in *S. calcitrans* following an interrupted meal, none was detected in the blood from the glass feeder. One possible explanation is that the amount of blood retained in the mouthparts, estimated at approximately 0.4 nL [35] was probably insufficient for transmission and/or detection of the bacteria in the blood from the glass feeder. Furthermore, the mouthparts are considered an unfavourable environment for pathogens due to the risk of desiccation or exposure to saliva [19, 35].

In the context of a complete blood meal, despite the detection of *A. phagocytophilum* DNA in most stable flies tested (29/30), with a relatively low mean Ct value of 23 at 24 h following a full blood meal, no bacterial DNA was detected in the blood in the glass feeder at any of the follow-up points. Similarly, the absence of *A. phagocytophilum* RNA in the glass feeder’s blood at 24 h was observed. This result was associated with a 50% decrease in the proportion of stable flies positive for *A. phagocytophilum* RNA. Regurgitation is rare in *S. calcitrans* in natural conditions [14]. Consequently, the absence or insufficient quantity of regurgitated infected blood (approximately 2 μL) [5] by *S. calcitrans* could explain why *A. phagocytophilum* DNA was not detected by qPCR, even after preamplification in the blood in the glass feeder. The increase in Ct values and the decrease of positive *S. calcitrans* for *A. phagocytophilum* DNA and RNA at 48 h and 24 h respectively, corresponding to bacterial degradation caused by the fly digestion, could be explained by the digestion of the blood meal in the midgut. During this digestion in the anterior midgut, antimicrobial peptides, such as defensins Smd1 and Smd2 or stomoxyn, are present [6, 16]. As part of the insect immune system, these peptides have a broad spectrum of activity, affecting the growth of microorganisms [12, 16]. For example, it has been demonstrated that stomoxyn is active against numerous Gram-negative and Gram-positive bacteria [6]. Moreover, the presence of *A. phagocytophilum* DNA in faeces as early as 1 h after an infected blood meal ingestion indicates that at least some of the blood is rapidly digested.

The density of flies around animals is another factor that influences mechanical transmission [1]. In total, 300 flies were used for each model, but in the field, high levels of infestation up to 1,000 stable flies per animal [31] can be observed during the vector activity season. Therefore, it cannot be ruled out that the absence of *A. phagocytophilum* DNA in the blood sampled from the glass feeder was due to an insufficient number of flies.

Regarding the field, the occurrence and efficiency of pathogen transmission by *S. calcitrans* could be affected by several factors, such as the density of *S. calcitrans* in the vicinity of animals, the presence of hosts in close proximity, the degree of bacteraemia in the hosts’ blood, and the proportion of infected and non-infected hosts as well as their proximity to each other. Furthermore, *S. calcitrans* can directly affect the host by encouraging close gathering of animals and by inducing stress and immunosuppression, thereby facilitating pathogen transmission [1].

In conclusion, the present study is the first to experimentally demonstrate the presence of *A. phagocytophilum* DNA and RNA in *S. calcitrans* for at least 24 h. Further investigations of the presence of *A. phagocytophilum* in stable flies in field conditions is worth considering. While current evidence suggests that *S. calcitrans* is unlikely to play a major role in the transmission of *A. phagocytophilum*, the minimum number of flies required for potential transmission remains unknown. Future experiments using both interrupted and complete feeding models with larger numbers of flies and triggering of regurgitation could help clarify whether *S. calcitrans* is capable of transmitting *A. phagocytophilum* to susceptible hosts or not. Nevertheless, these results are reassuring in terms of the risk of *A. phagocytophilum* transmission. They suggest that *S. calcitrans* play, at worst, a minor role in transmitting *A. phagocytophilum* within a cattle herd, and an even smaller role between herds. Furthermore, the present study promotes the use of innovative *ex vivo* vector infection models as alternatives to animal-based models, in compliance with animal welfare regulations and 4R principles.

## Supplementary material

*Supplementary Table 1*. DNA and RNA detection of *Anaplasma phagocytophilum* in blood used for the interrupted and complete model at H0.

*Supplementary Table 2*. Detection of *Anaplasma phagocytophilum* DNA and RNA at different times in *Stomoxys calcitrans* after a complete meal of infected blood.

The supplementary material of this article is available at https://www.parasite-journal.org/10.1051/parasite/2026021/olm.
