# Supplementary material for: Stomoxys calcitrans as a potential mechanical vector of Anaplasma phagocytophilum: assessment through original ex vivo feeding models
Source: Parasite. 2026 Apr 22;33:25. doi: 10.1051/parasite/2026021 (PMC13105450; doi:10.1051/parasite/2026021)
Supplement: Supplementary file 1 — Supplementary Table 1. DNA and RNA detection of Anaplasma phagocytophilum in blood used for the interrupted and complete model at H0. [file parasite-33-25-s1.pdf]

Supplementary Table 1. DNA and RNA detection of *A. phagocytophilum* in blood used for interrupted and complete model at H0

	DNA detection (C_T values)	RNA detection (C_T values)		DNA detection (C_T values)	RNA detection (C_T values)
**Experiments**	**H0**	**H0**	**Experiments**	**H0**	**H0**
**IntM1**	16.04	26.66	**ComM4**	13.94	N.D.
**IntM2**	14.35	20.93	**ComM5**	14.61	N.D.
**IntM3**	14.61	22.34	**ComM6**	15.70	N.D.
**IntM4**	14.59	*N.D.*	**ComM1**	17.13	27.07
**IntM5**	15.23	*N.D.*	**ComM2**	16.31	22.11
**IntM6**	14.00	*N.D.*	**ComM3**	17.20	22.25

N.D. not determined

IntM: Interrupted blood meal

ComM: Complete blood meal
