# Supplementary material for: Stomoxys calcitrans as a potential mechanical vector of Anaplasma phagocytophilum: assessment through original ex vivo feeding models
Source: Parasite. 2026 Apr 22;33:25. doi: 10.1051/parasite/2026021 (PMC13105450; doi:10.1051/parasite/2026021)
Supplement: Supplementary file 2 — Supplementary Table 2. Detection of Anaplasma phagocytophilum DNA and RNA at different times in Stomoxys calcitrans after a complete meal of infected blood. [file parasite-33-25-s2.pdf]

**Supplementary Table 2. Detection of *A. phagocytophilum* DNA and RNA at different times in *S. calcitrans* after a complete meal of infected blood.**

**Table 2A.** Mean CT values of *A. phagocytophilum* DNA (without pre-amplification) at different times in *S. calcitrans* after a complete meal of infected blood. Ten *S. calcitrans* were tested per time and per experiment (n=3) and the mean CT ± SEM was calculated on positive *S. calcitrans*.

| **Experiments** | **ComM4** | | **ComM5** | | **ComM6** | | **Total** | |
|---|---|---|---|---|---|---|---|---|
| **Time** | Mean CT ¤ ± SEM | Positive *S. calcitrans* for Aph | Mean CT ¤ ± SEM | Positive *S. calcitrans* for Aph | Mean CT ¤ ± SEM | Positive *S. calcitrans* for Aph | Mean CT ¤ ± SEM | Positive *S. calcitrans* for Aph |
| **H1** | **27.11**±2.26 | 8/10 | **19.67**±0.17 | 10/10 | **19.57**±0.43 | 10/10 | **21.76**±0.91 | 28/30 |
| **H2** | **25.81**±1.87 | 9/10 | **23.34**±1.79 | 10/10 | **19.12**±0.35 | 10/10 | **22.65**±0.97 | 29/30 |
| **H3** | **23.97**±2.02 | 10/10 | **22.06**±1.71 | 10/10 | **18.64**±0.13 | 10/10 | **21.55**±0.95 | 30/30 |
| **H6** | **24.07**±2.21 | 10/10 | **21.64**±1.59 | 10/10 | **18.45**±0.09 | 10/10 | **21.39**±0.97 | 30/30 |
| **H9** | **20.40**±1.23 | 10/10 | **21.12**±1.55 | 9/10 | **20.32**±1.50 | 10/10 | **20.59**±0.80 | 29/30 |
| **H12** | **18.86**±0.26 | 10/10 | **23.98**±2.41 | 9/10 | **20.18**±1.53 | 10/10 | **20.90**±0.97 | 29/30 |
| **H24** | **19.84**±0.27 | 10/10 | **25.87**±1.88 | 9/10 | **24.31**±1.86 | 10/10 | **23.25**±0.97 | 29/30 |
| **H48*** | **29.54**±1.08 | 6/10 | **32.84**±1.80 | 8/10 | **32.47**±1.75 | 7/10 | **31.77**±0.96 | 21/30 |
| **H72*** | **33.32**±0.78 | 10/10 | - | 0/10 | **34.31**±1.62 | 3/10 | **33.55**±0.68 | 13/30 |
| **H96*** | **34.07**±0.54 | 2/10 | - | 0/10 | - | 0/10 | **34.07**±0.54 | 2/30 |
| **H120*** | **35.77**±0.09 | 2/10 | - | 0/10 | **35.67** | 1/10 | **35.73**±0.06 | 3/30 |

¤Mean CT on positive *S. calcitrans* for *A. phagocytophilum*

* Flies were fed with non-infected blood every 24h starting 48h after the initial blood meal

Aph: *A. phagocytophilum*

ComM: Complete blood meal

**Table 2B.** Mean CT values of *A. phagocytophilum* RNA (with pre-amplification) at different times in *S. calcitrans* after a complete meal of infected blood. Ten *S. calcitrans* were tested per time and per experiment (n=3) and the mean CT ± SEM was calculated on positive *S. calcitrans*.

| **Experiments** | **ComM1** | | **ComM2** | | **ComM3** | | **Total** | |
|---|---|---|---|---|---|---|---|---|
| **Time** | Mean CT ¤ ± SEM | Positive *S. calcitrans* for Aph | Mean CT ¤ ± SEM | Positive *S. calcitrans* for Aph | Mean CT ¤ ± SEM | Positive *S. calcitrans* for Aph | Mean CT ¤ ± SEM | Positive *S. calcitrans* for Aph |
| **H1** | **17.21**±0.27 | 8/10 | **15.97**±0.12 | 8/10 | **16.57**±0.25 | 10/10 | **16.58**±0.16 | 26/30 |
| **H2** | **18.27**±0.60 | 9/10 | **16.85**±1.00 | 9/10 | **16.13**±0.12 | 9/10 | **17.08**±0.41 | 27/30 |
| **H3** | **17.42**±0.59 | 10/10 | **16.51**±0.70 | 6/10 | **16.21**±0.11 | 9/10 | **16.77**±0.30 | 25/30 |
| **H6** | **18.28**±0.37 | 7/10 | **16.05**±0.22 | 10/10 | **19.75**±1.52 | 8/10 | **17.86**±0.58 | 25/30 |
| **H9** | **19.51**±0.58 | 8/10 | **17.65**±0.56 | 8/10 | **17.99**±0.46 | 10/10 | **18.35**±0.33 | 26/30 |
| **H12** | **22.32**±0.78 | 9/10 | **18.85**±0.58 | 9/10 | **18.95**±0.42 | 7/10 | **20.13**±0.49 | 25/30 |
| **H24** | **23.28**±1.20 | 3/10 | **22.38**±0.37 | 6/10 | **21.10**±0.39 | 6/10 | **22.05**±0.36 | 15/30 |
| **H48*** | - | 0/10 | - | 0/10 | - | 0/10 | - | 0/10 |
| **H72*** | - | 0/10 | - | 0/10 | - | 0/10 | - | 0/10 |

¤Mean CT on positive *S. calcitrans* for *A. phagocytophilum*

*flies were fed with non-infected blood every 24h starting 48h after the initial blood meal

Aph: *A. phagocytophilum*

ComM: Complete blood meal
